# Augmentation of spinal cord glutamatergic synaptic currents in zebrafish primary motoneurons expressing mutant human *TARDBP* (TDP-43)

**DOI:** 10.1038/s41598-019-45530-3

**Published:** 2019-06-24

**Authors:** Virginie Petel Légaré, Ziyaan A. Harji, Christian J. Rampal, Xavier Allard-Chamard, Esteban C. Rodríguez, Gary A. B. Armstrong

**Affiliations:** Department of Neurology and Neurosurgery, Montreal Neurological Institute, Faculty of Medicine, McGill University, Montreal, Canada

**Keywords:** Amyotrophic lateral sclerosis, Neurological disorders, Amyotrophic lateral sclerosis

## Abstract

Though there is compelling evidence that de-innervation of neuromuscular junctions (NMJ) occurs early in amyotrophic lateral sclerosis (ALS), defects arising at synapses in the spinal cord remain incompletely understood. To investigate spinal cord synaptic dysfunction, we took advantage of a zebrafish larval model and expressed either wild type human *TARDBP* (wt*TARDBP*) or the ALS-causing G348C variant (mut*TARDBP*). The larval zebrafish is ideally suited to examine synaptic connectivity between descending populations of neurons and spinal cord motoneurons as a fully intact spinal cord is preserved during experimentation. Here we provide evidence that the tail-beat motor pattern is reduced in both frequency and duration in larvae expressing mut*TARDBP*. In addition, we report that motor-related synaptic depolarizations in primary motoneurons of the spinal cord are shorter in duration and fewer action potentials are evoked in larvae expressing mut*TARDBP*. To more thoroughly examine spinal cord synaptic dysfunction in our ALS model, we isolated AMPA/kainate-mediated glutamatergic miniature excitatory post-synaptic currents in primary motoneurons and found that in addition to displaying a larger amplitude, the frequency of quantal events was higher in larvae expressing mut*TARDBP* when compared to larvae expressing wt*TARDBP*. In a final series of experiments, we optogenetically drove neuronal activity in the hindbrain and spinal cord population of descending ipsilateral glutamatergic interneurons (expressing *Chx10*) using the Gal4-UAS system and found that larvae expressing mut*TARDBP* displayed abnormal tail-beat patterns in response to optogenetic stimuli and augmented synaptic connectivity with motoneurons. These findings indicate that expression of mut*TARDBP* results in functionally altered glutamatergic synapses in the spinal cord.

## Introduction

While most forms of ALS occur sporadically in the population (sALS), a small subset (~5–10%) with the same cardinal symptoms and progression is inherited (fALS). A breakthrough came in 2006 with the discovery that TDP-43 is a major component of ubiquitin-positive tau-negative cytoplasmic neuronal aggregates with clearance of nuclear TDP-43 occurring in the majority (>90%) of ALS cases^1^. Subsequent genetic sequencing identified dozens of mutations in the gene encoding TDP-43 (*TARDBP*) that account for a small percentage of both sALS cases (~1%) and dominantly inherited fALS cases (3–4%)^2,3^. TDP-43 is a ubiquitously expressed member of the heterogeneous nuclear ribonucleoprotein family of proteins. Though its biological function is not fully understood, TDP-43 is involved in several steps of RNA metabolism, and evidence for gene transcription^4^, splicing^5,6^ and autoregulation of its own cellular expression levels^7–10^ has been reported. Previous work examining RNA targets processed by TDP-43 have identified transcripts associated with synapse formation, regulation of neurotransmitter signaling, and neuronal development^8,11–15^. As with a mouse knockout model^16^, zebrafish Tdp-43 loss of function models are developmentally lethal and display impaired NMJ synaptic function prior to death^17–20^. Investigations of the pathophysiological basis of altered synaptic function in the spinal cord of animal models expressing ALS-associated TDP-43 variants remains a poorly explored area of research yet would be useful for identifying cellular pathways involved in motoneuron dysfunction.

Corticomotoneuronal dysfunction in ALS was initially proposed by Charcot^21^, but it was not until the advent of recording motor-evoked potentials in muscles following transcranial magnetic stimulation of the motor cortex in symptomatic patients, that evidence of cortical hyperexcitability with reduced intracortical inhibition was identified as an early (and possibly pre-symptomatic)^22^ pathophysiological feature of ALS^22–27^. There remains considerable debate about the underlying cause of cortical hyperexcitability in ALS^28^, and evidence of both neuronal intrinsic (*e.g*. increased persistent Na^+^ currents in motoneurons, altered AMPA receptor subunit composition) and extrinsic (*e.g*. increased glutamatergic synaptic activity) factors have been reported in mouse *SOD1*^G93A^ cortical cultures^29,30^ and *in vitro* slice recordings of pyramidal motoneurons^31^. What role cortical hyperexcitability plays in the degenerative process of upper motoneurons, along with the consequences of increased excitatory glutamatergic synaptic output to the lower spinal motor network, remains to be fully understood. However, evidence from a rat *SOD1* model suggests that targeted knockdown of mutant *SOD1* in the motor cortex alone can extend the lifespan, delay lower motoneuron degeneration, and maintain NMJs^32^. Studies taking a similar approach using transgenic *TARDBP* models have yet to be undertaken. Despite the proposed role excitotoxicity has in the pathogenesis of ALS, *in vivo* experiments examining glutamatergic synaptic transmission in the spinal cord have been a challenge to perform as slice preparations of murine spinal cords severs descending synaptic inputs to ventral horn motoneurons.

Using zebrafish, an aquatic vertebrate, which lends easily to investigations of spinal cord synaptic function, we examined *in vivo* spinal cord excitatory synaptic defects arising following the expression of the ALS-associated *TARDBP* missense mutation 1176G > T (encoding the G348C variant). This model was previously shown to have impairments in both touch-evoked locomotor behaviour and synaptic transmission at the NMJ^33–36^. Here, we report findings demonstrating that descending excitatory glutamatergic synaptic currents in spinal cord primary motoneurons are increased in amplitude lending support to the theory that in ALS spinal cord motoneurons are subjected to increased and possibly deleterious excitatory inputs.

## Methods

### Zebrafish lines

Wild type (TL) zebrafish (*Danio rerio*) were bred and maintained according to standard procedures^37^. This study was performed in accordance with the guidelines of the Canadian Council for Animal Care and conducted at the Research Centre of the University of Montréal Hospital Centre. In addition, we confirm that the experimental protocols were approved by the animal care committee of the Research Centre of the University of Montréal Hospital Centre. To obtain transgenic zebrafish larvae expressing *ChRWR-EGFP* in *Chx10* expressing neurons, Tg(*UAS:ChRWR-EGFP*) zebrafish were crossed with Tg(*Chx10:Gal4*) zebrafish. In some experiments transgenic larvae expressing *Hb9:GFP* were used for preparing example images of spinal cord motoneurons. All experiments were performed on sexually undifferentiated zebrafish larvae 48–56 hpf.

### Preparation and Injection of *TARDBP* mRNA

Human *TARDBP* cDNA was obtained from Open Biosystems. The mutation encoding the G348C variant was introduced using site-directed mutagenesis in the appropriate vector using QuikChange XL Site-Directed Mutagenesis Kit (Stratagene) as previously described^36^. Constructs encoding N-FLAG and C-Myc were incorporated and subcloned into pCS2+ plasmid vectors which were subsequently used to generate mRNA.

Injections in 1 cell stage blastulae were performed as previously described^33,36^. Briefly, wt*TARDBP* and mut*TARDBP* (G348C) mRNAs were transcribed from *Not*I-linearized pCS2+ using SP6 polymerase with the mMESSAGE Machine Kit (Ambion). The mRNA was diluted in nuclease free water (Ambion) with 0.05% Fast Green (Sigma) to a final concentration of 25 ng/µl and backfilled in a pulled (Sutter instrument company) thin-walled borosilicate capillary tube and pressure injected into the cell using a PicoSpritzer III (General Valve). No obvious gross anatomical disparity in larval body shape was observed across treatments at 54 hpf, nor was there any apparent delay in development, as migration of the lateral line primordium (used for staging development)^38^ was unaffected by exogenous mRNA expression.

### Evoked tail-beat behaviour

Touch-evoked assessment of larval tail-beat patterns was performed at room temperature (22–25 °C) in larvae aged 54 hpf. Larvae were suspended in a dish containing 1% solution low melting point agarose (Sigma). After the gel was set, the agarose around the larval tail was removed, liberating the tail and allowing for an unobstructed tail-beat pattern following a light touch with a pair of forceps. Tail-beat patterns were recorded digitally at 200 frames/sec (Grasshopper 2 camera, Point Grey Research) and subsequently analysed using ImageJ software. Optogenetically-evoked tail-beat patterns of activity were similarly obtained by embedding larvae in agarose and exposing larvae to blue light from a wide-field fluorescence light source (X-Cite 120Q, Excelitas technologies) at an intensity of 0.4 mW/mm^2^.

### Whole-cell voltage clamp recordings

As described previously^39^, zebrafish were anaesthetized in 0.04% tricaine (Sigma) dissolved in modified Evan’s solution containing, in mM: 134 NaCl, 2.9 KCl, 2.1 CaCl_2_, 1.2 MgCl_2_, 10 HEPES, 10 glucose. The solution was adjusted to 290 mOsm and a pH of 7.8. The zebrafish were then restrained with fine (0.001 in.) tungsten wires pierced through their notochords and secured to a Sylgard-lined dish. The outer layer of skin between the pins was removed using a fine glass electrode and forceps, exposing the musculature. The preparation was then visualized by oblique illumination (Olympus BX61W1). For motoneuron recordings, collagenase (1 mg/ml) was perfused over the preparation for 10 min. This allowed for partial digestion before muscle cells overlying the spinal cord were removed by aspiration to expose the spinal cord, while leaving the ventral root and deeper ventral muscle cells intact.

Standard whole-cell voltage clamp or current clamp recordings were obtained from the CaP motoneuron or fast twitch trunk muscle cells in somites 13–16. 8–10 MΩ glass electrodes were pulled from thin-walled Kimax-51 borosilicate glass (Kimble Chase) and filled with the following intracellular solution (in mM: 116 K-gluconate; 16 KCl; 2 MgCl_2_; 10 HEPES; 10 EGTA; adjusted to pH 7.2, 290 mOsm). Muscle cells and neurons were held near their resting potential at −65 mV and series resistance was <15 MΩ and compensated to 70–90%. Cells with resting membrane potentials less then −55 mV were not used. In touch-evoked motoneuron recordings, 15 µM tubocurarine (Sigma) was used to block NMJ synaptic transmission. In recordings of optogenetically-evoked glutamatergic excitatory post-synaptic currents (EPSCs) in CaP motoneurons, 100 µM bicuculline (Sigma) and 5 µM strychnine (Sigma) were perfused over the preparation and baseline voltage recordings were adjusted to zero to facilitate EPSC event detection. In recordings of quantal currents, 1 µM TTX (Sigma), 50 µM DL-2-amino-5-phosphonovaleric acid (APV, Sigma), 100 µM bicuculline (Sigma) and 5 µM strychnine (Sigma) were perfused over the preparation to isolate AMPA/kainate-mediated miniature excitatory post-synaptic currents (mEPSC). All electrophysiological data were sampled at 50 kHz using an Axopatch 200B amplifier (Molecular Devices) or BVC-700A amplifier (Dagan), digitized using a Digidata 1440A (Molecular Devices) and stored on a computer for later analysis using pCLAMP 10 software (Molecular Devices).

### Western blots

Larvae aged 54 hpf were deyolked using Ginsburg Fish Ringer (NaCl, KCl, NaHCO_3_) solution. For each condition, 40 larvae were homogenized using 1x RIPA buffer (EMD Millipore RIPA lysis Buffer 10×) and protease inhibitor cocktail (cOmplete^™^, Mini, EDTA-free Protease Inhibitor Cocktail). Lysate was recovered following centrifugation. For each sample, protein was quantified using a Bradford assay, and 35 μg at equal concentration were loaded on 12.5% SDS polyacrylamide gel. Separated proteins were transferred to a nitrocellulose membrane and blocked in 5% milk (TSBT) for 1 hr at room temperature. Membrane was incubated with myc primary antibody (monoclonal, Cell Signaling Technology (9b11), 1:1000) in blocking buffer at 4 °C overnight. Mouse secondary antibody (Jackson Immuno, 1:10 000) was applied for 1 hour at room temperature, and enhanced chemiluminescence was used for visualization. Following stripping using a mild stripping buffer (Glycine, SDS, Tween 20, pH 2.2), membrane was re-probed with Actin (C4 MPBio, 1: 10 000), and visualised using the same method as previously described.

### Image acquisition

Images of larval trunk and spinal cords were visualized using a Quorum Technologies spinning disk confocal microscope with CSU10B (Yokogawa) spinning head mounted on an Olympus BX61W1 fluorescence microscope and connected to a Hamamatsu ORCA-ER camera. Images were acquired using Volocity software (Quorum Technologies).

### Statistical analysis

SigmaPlot 11.0 integrated with SigmaStat 3.1 was used to assess data groupings for significance. Statistical analyses used one-way repeated measures ANOVA, followed by a post-hoc Tukey multiple comparison test. Significance was assessed at *p* ≤ 0.05 (asterisks, comparison to wild type larvae) or *p* ≤ 0.05 (dagger, comparison to wt*TARDBP* expressing larvae).

## Results

### Expression of mut*TARDBP* impairs locomotor tail-beat pattern

**Figure 1:**
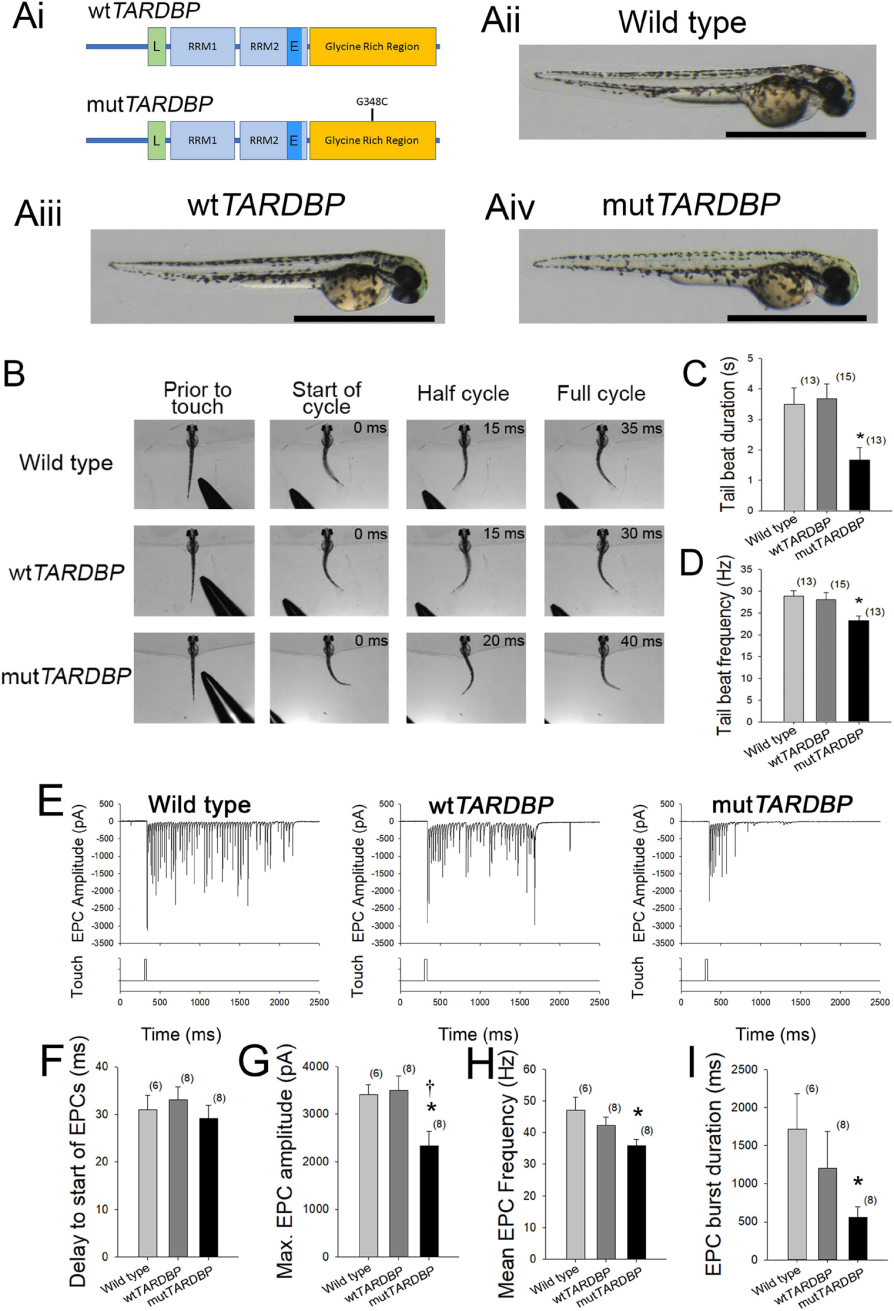
Expression of mut*TARDBP* results in touch-evoked defects in locomotor pattern generation. (**Ai**), Schematic representation of human wild type *TARDBP* and mutant *TARDBP* encoding the ALS-causing missense mutation G348C situated in the C-terminal glycine rich region. Other general structures of *TARDBP* include an N-terminal nuclear localization motif (L), RNA binding domains (RRM1 and RRM2) and a proposed nuclear export motif (E). (**Aii–Aiv**), Representative images of 52–54 hpf zebrafish larvae. Scale bar represents 1 mm. (**B)** Example still frames from high speed video recordings of touch-evoked locomotor tail-beat patterns in zebrafish larvae. Tail-beat patterns were found to be reduced in tail beat duration (**C**) and mean frequency (**D**) when compared to either wild type zebrafish or zebrafish larvae expressing wt*TARDBP*. Defects in pattern generation were observed in the synaptic inputs to muscle cells of the trunk during fictive locomotor patterns. (**E**) Example traces of whole-cell patch clamp recordings of rhythmic fast-twitch EPCs evoked by a light touch to the tail. No differences were found between the tail touch and the start of the EPCs (**F**), but the maximum EPC amplitude (**G**), mean EPC frequency (**H**) as well as the EPC burst duration (**I**) were all found to be reduced in larvae expressing mut*TARDBP*. Numbers in parentheses represent sample sizes. Asterisks and daggers represent statistical differences from wild type (*p* < 0.05) and wt*TARDBP* (*p* < 0.05) larvae respectively.

Transient expression of mut*TARDBP* in zebrafish larvae aged 52–56 hours post fertilization (hpf) (Fig. 1A,B) has been previously shown to result in reduced touch-evoked locomotor swim duration, velocity and distance when compared to larvae expressing wild type *TARDBP* (wt*TARDBP*) mRNA^33,34^ despite having similar protein expression levels (Supplementary Material, Fig. S1). To gain further insight into the underlying motor defect arising in larvae expressing mut *TARDBP,* touch-evoked locomotor tail-beat patterns were video recorded in animals partially embedded in agarose and aged 54 hpf (Supplementary Material, Video S1, Fig. 1C). No differences in tail-beat frequency or tail-beat duration were found between wild type larvae and larvae expressing wt*TARDBP* (Fig. 1C). However, larvae expressing mut*TARDBP* displayed both a reduction in tail-beat duration (*p* < 0.04) and tail-beat frequency (*p* < 0.05) when compared to either wild type larvae or zebrafish larvae expressing wt*TARDBP* (Fig. 1C,D). Tail-beat durations recorded across all three treatment groups were lower than previously reported touch-evoked swim durations in unrestrained (freely swimming) larvae expressing the same constructs^33^, and may have resulted from reduced sensory feedback from hair cells of the anterior lateral line^40^ due to the partial embedding of the larvae.

### Mut*TARDBP* expression results in attenuated rhythmic muscle endplate currents (EPCs)

To investigate where defects in pattern generation arise in larvae expressing mut*TARDBP*, whole-cell voltage clamp recordings of touch-evoked rhythmic muscle endplate currents (EPCs) were recorded from fast-twitch muscle cells of the trunk (Fig. 1E) using previously established techniques and held at −65 mV^41^. At 48 hpf, each side of the larval zebrafish trunk muscular system is composed of 32 hemisomites with each somite containing a superficial slow-twitch muscle cell layer followed by three layers of fast-twitch muscles cells that are polyinnervated by 3 primary and around 20 secondary motoneurons. No difference in the delay between touching the tail and the start of rhythmic muscle EPCs was found among the treatment groups suggesting that the sensory system of larvae expressing mut*TARDBP* is not impaired (Fig. 1F). Not surprisingly, the maximum touch-evoked EPC amplitude was found to be reduced in larvae expressing mut*TARDBP* when compared to either wt*TARDBP* or wild type larvae (*p* < 0.05; Fig. 1G). This finding likely reflects previously reported presynaptic impairment of synaptic transmission at the NMJ and not a muscle-specific defect^33,34^. In addition, both mean frequency and burst duration of rhythmic EPCs were found to be reduced in larvae expressing mut*TARDBP* when compared to muscle recordings from wild type larvae (*p* < 0.05; Fig. 1H,I) but not larvae expressing wt*TARDBP*. This observation suggests that in addition to synaptic transmission defects at the NMJ, defects in the circuitry coordinating motor behaviour is altered in larvae expressing mut*TARDBP*.

### Primary motoneurons expressing mut*TARDBP* display reduced durations of motor-related depolarizations

**Figure 2:**
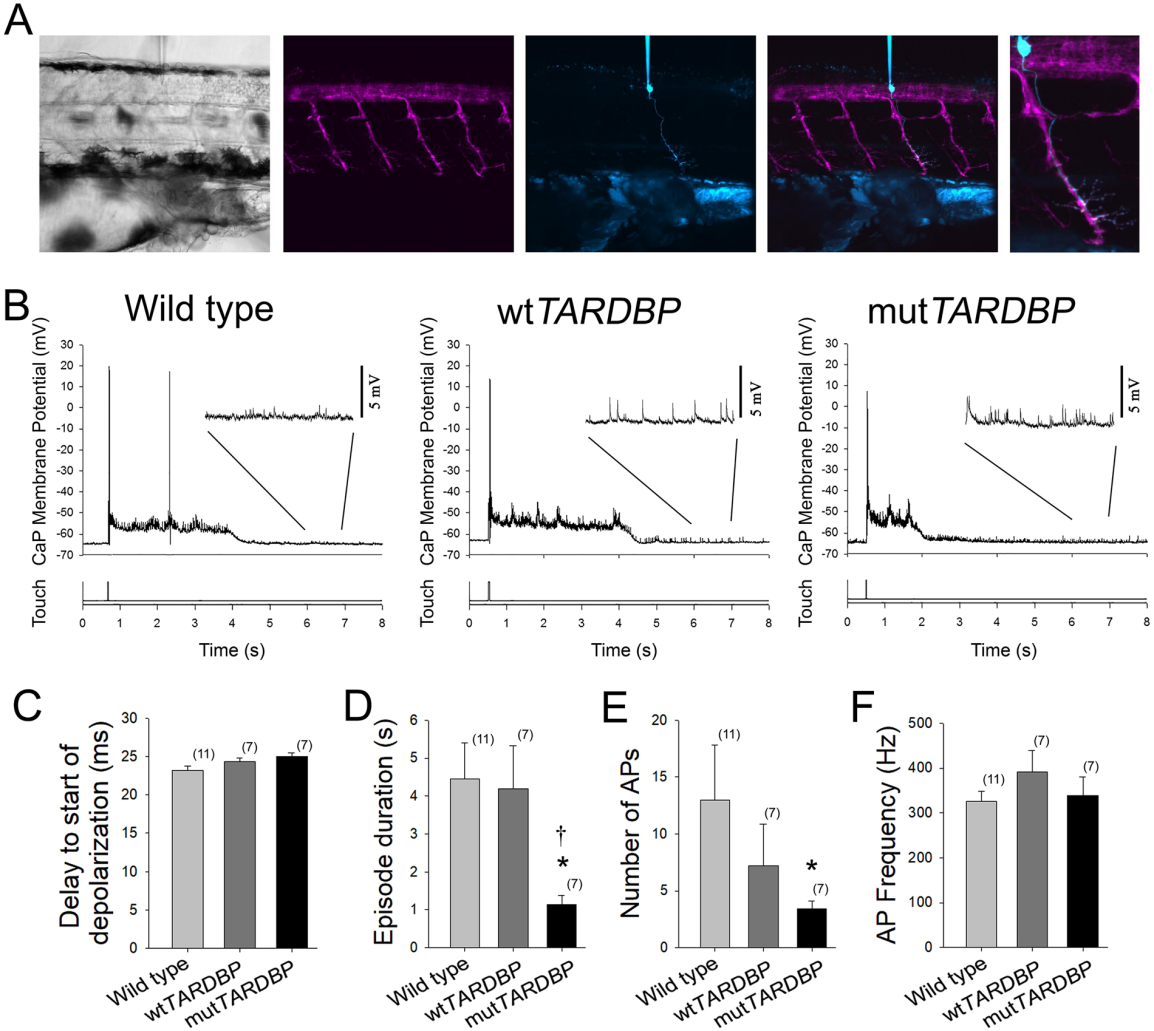
Attenuation of fictive synaptic depolarizations to spinal primary motoneurons during touch-evoked locomotor activity in larvae expressing mut*TARDBP*. (**A**) From left to right: a bright field image of a 54 hpf zebrafish trunk in a transgenic larvae expressing GFP (magenta) under the control of the motoneuron specific promotor *Hb9*, with a patch electrode dialyzing a CaP motoneuron with sulforhodamine (cyan). Fictive motor patterns were elicited with a light puff of water onto the tail using a picospritzer. (**B**) Example traces of CaP motoneuron synaptic depolarizations following light touch. Note the presence of increased spontaneous EPSPs following touch-evoked motor-related depolarizations (insets). (**C**) No differences were found in the delay between the touch and start of depolarizations among wild type larvae, larvae expressing wt*TARDBP* or larvae expressing mut*TARDBP*. However, both the duration of synaptic depolarizations (**D**) and the number of APs (**E**) elicited following light touch in larvae expressing mut*TARDBP* were found to be reduced when compared to either wild type larvae or larvae expressing wt*TARDBP*. Though fewer APs were generated in larvae expressing mut*TARDBP*, the frequency of APs (**F**) was not found to be different from wild type larvae or wt*TARDBP* larvae. Numbers in parentheses represent sample sizes. Asterisks and daggers represent statistical differences from wild type (*p* < 0.05) and wt*TARDBP* (*p* < 0.05) larvae respectively.

To investigate where defects in motor pattern generation originate, whole-cell current clamp recordings of touch-evoked patterns of motor-related activity were recorded in primary motoneurons. The caudal and primary (CaP) motoneuron^42^ is one of three primary motoneurons located in each hemisomite of the spinal cord and is easily identified by its ventral position in the spinal cord and large cell body with distinctive ventral axon projection and is labelled by the Tg[*Hb9:GFP*] line^43^ (Fig. 2A). As this neuron has been previously used to investigate NMJ synaptic defects^33^, and to reduce the variability that likely exists within the motoneuron population, it was used here to examine fictive motor activity patterns following a light touch to the tail in immobilized larvae (Fig. 2B). Similar to the findings in touch-evoked rhythmic muscle EPC recordings, no difference between the delay and start of touch-evoked CaP depolarizations was found among the experimental groups (Fig. 2B,C). At this stage in zebrafish development, motoneuron depolarizations are mediated by a primarily tonic depolarizing glycinergic-mediated synaptic current^44^ driven by developmentally reversed high intracellular concentration of Cl^−^ in the vertebrate nervous system^45–48^ and rhythmic glutamatergic synaptic input to spinal motoneurons^49,50^. Mean touch-evoked membrane depolarization of the CaP membrane potential was not found to differ among the experimental groups (wild type, 6.5 ± 0.7 mV; wt*TARDBP*, 6.2 ± 1.2 mV; mut*TARDBP* 5.9 ± 0.4 mV), but the duration of the touch-evoked episode duration was found to be reduced in CaP motoneurons expressing mut*TARDBP* (p < 0.05, Fig. 2D) when compared to recordings in either wild type or wt*TARDBP* expressing CaP motoneurons. In addition, fewer action potentials (APs) were generated (p < 0.05, Fig. 2E) in zebrafish larvae expressing mut*TARDBP* compared to APs generated in wild type CaP motoneurons. Despite the reduction in the number of evoked APs observed in CaP motoneurons expressing mut*TARDBP* the mean frequency of APs was not found to differ among the experimental groups (Fig. 2F).

### Quantal glutamatergic mEPSCs occur at a higher frequency and are scaled-up in primary motoneurons expressing mut*TARDBP*

**Figure 3:**
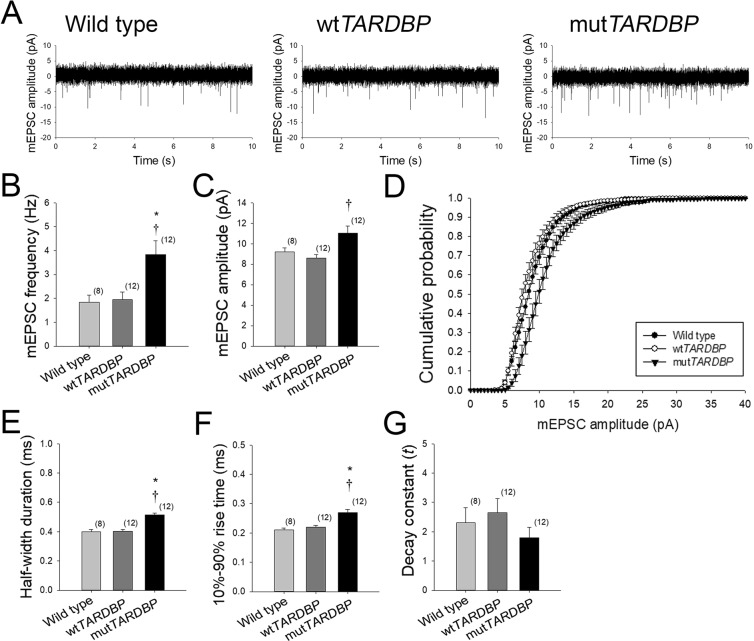
Synaptic scaling of AMPA-mediated glutamatergic mEPSCs in primary motoneurons expressing mut*TARDBP*. (**A**) Example 10 second recordings of quantal AMPA-mediated glutamatergic mEPSCs recorded in CaP motoneurons. AMPA-mediated glutamatergic mEPSCs in mut*TARDBP* larvae were found to occur at an increased frequency when compared to either wild type larvae or larvae expressing wt*TARDBP* (**B**). The amplitude of mEPSCs recorded in CaP motoneurons expressing mut*TARDBP* were found to be significantly larger in amplitude when compared to larvae expressing wt*TARDBP* (**C**). Furthermore, when the entire recorded population of mEPSCs was examined, the cumulative probability of AMPA-mediated quantal events in larvae expressing mut*TARDBP* was found to be skewed right, indicating synaptic scaling (**D**). In addition, the half-width duration (**E**) and the rise time (**F**) of quantal events in larvae expressing mut*TARDBP* were found to be increased but no difference in the decay constant (**G**) was evident. Numbers in parentheses represent sample sizes. Asterisks and daggers represent statistical differences from wild type (*p* < 0.05) and wt*TARDBP* (*p* < 0.05) larvae respectively.

A general observation made in recordings of touch-evoked activity in CaP motoneurons expressing mut*TARDBP*, and to a lesser extent wt*TARDBP*, was a persistent increase in spontaneous synaptic depolarizations following locomotor-related membrane depolarizations (Fig. 2B insets). To further investigate the nature of these spontaneous events, recordings of AMPA/kainate-mediated glutamatergic miniature excitatory post-synaptic currents (mEPSCs) were made in CaP motoneurons (Fig. 3A). In 10 minute recordings of AMPA/Kainate mEPSCs, larvae expressing mut*TARDBP* were strikingly found to display an increase in the frequency of events (*p* < 0.01, Fig. 3B) when compared to quantal events recorded in either wild type or wt*TARDBP* expressing CaP motoneurons. The mean amplitude of mEPSCs was also found to be larger in CaP motoneurons expressing mut*TARDBP* (*p* < 0.01, Fig. 3C) when compared to larvae expressing wt*TARDBP* but not mEPSCs recorded in wild type CaP motoneurons. When the entire population of mEPSC amplitudes was plotted as a cumulative histogram, a right-shifted skewing of mEPSC amplitudes in CaP motoneurons expressing mut*TARDBP* was evident (Fig. 3D), indicating synaptic scaling of glutamatergic synaptic strength in spinal motoneurons expressing mut*TARDBP*. This increase in mEPSC amplitude was also accompanied by an increase in half-width duration (p < 0.01, Fig. 3E) and an increased quantal rise time (10–90% of amplitude) of events (p < 0.01, Fig. 3F), but not a change in the decay constant (Fig. 3G) in CaP motoneurons expressing mut*TARDBP* when compared to mEPSCs recorded in wild type larvae or larvae expressing wt*TARDBP*.

### Augmentation of glutamatergic synaptic strength in primary motoneurons expressing mut*TARDBP*

**Figure 4:**
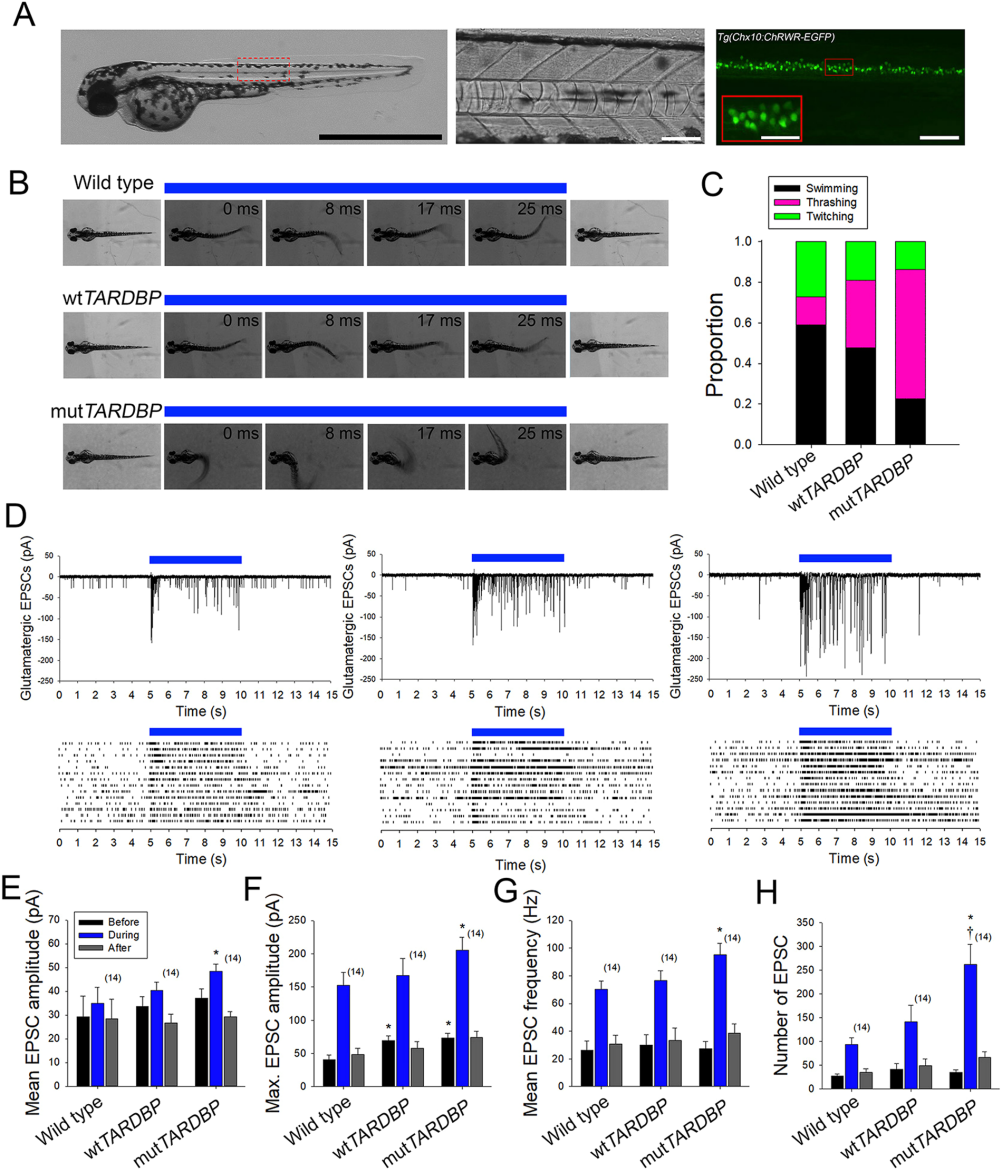
Augmentation of descending excitatory glutamatergic synaptic inputs to primary motoneurons expressing mut*TARDBP*. (**A**) Example bright field of image of a 56 hpf transgenic *Chx10:ChRWF-EGFP* zebrafish larva (left, scale bar represents 1 mm). Bright field image showing a section of the zebrafish trunk showing 6 somites (middle, scale bar represents 50 μm) with corresponding EGFP fluorescence image depicting spinal V2a interneurons expressing *Chx10:ChRWF-EGFP* (right, scale bar represents 50 μm and in inset 25 μm). (**B**) Example still frames from high speed video recordings of optogenetic stimulation of locomotor tail-beat patterns in zebrafish larvae partially embedded in agarose. (**C**) Proportion of larvae exhibiting different patterns of optogenetic-evoked activity, with the majority of larvae expressing mut*TARDBP* displaying tail thrashing. Sample sizes for optogenetically-evoked patterns of activity for wild type, wt*TARDBP* and mut*TARDBP* larval groups were 17, 21, and 22 respectively. (**D**) Example whole-cell voltage clamp recordings of optogenetically-evoked synaptic currents in CaP motoneurons (above) and EPSC raster plots from individual larvae (below). Recorded EPSC were binned into three groups, representing: 5 seconds before optogenetic stimulation, 5 seconds during light exposure, and the following 5 seconds after stimulation. Both the mean (**E**) and maximum amplitudes (**F**) of EPSCs recorded in CaP motoneurons in larvae expressing mut*TARDBP* were found to be higher than EPSCs recorded in wild type CaP motoneurons. Furthermore, the mean frequency of optogenetically driven EPSCs was found to be higher in larvae expressing mut*TARDBP* when compared to wild type EPSCs in CaP motoneurons (**G**). When the total number of evoked EPSCs was examined over the 5 second stimulus, larvae expressing mut*TARDBP* were found to generate more EPSCs than both wild type and wt*TARDBP* expressing larvae (**H**). Asterisks and daggers represent statistical differences from wild type (*p* < 0.05) and wt*TARDBP* (*p* < 0.05) larvae respectively.

To further investigate spinal glutamatergic defects, locomotor activity was evoked optogenetically in larvae expressing channelrhodopsin wide receiver variant^51^ in neurons expressing the transcription factor *Chx10*^52^ using the Gal4-UAS system (Tg(*Chx10:Gal4;UAS:ChRWR-EGFP*), Fig. 4A). The transcription factor *Chx10* is expressed in reticulospinal neurons of the larval zebrafish hindbrain and spinal cord that have ipsilateral descending axon projections and belong to the glutamatergic V2a class of interneurons^53^. These neurons form synaptic connections with spinal motoneurons and play a significant role in coordinating descending locomotor synaptic drive in the larval spinal cord^52–55^. Optogenetically-evoked locomotor tail-beat patterns were video recorded in animals aged 54 hpf and partially embedded in agarose with the tail free to move (Fig. 4B). Activity patterns were evoked by 5 second exposure to blue light and could be separated into three general groups: anguilliform tail-beat, tail thrashing, and slight twitching of the trunk in response to blue light. Both wild type and larvae expressing wt*TARDBP* displayed mainly anguilliform tail-beat patterns in response to optogenetic stimulation (Supplementary Material, Video S2, Fig. 4B,C). In contrast, optogenetically-evoked locomotor activity in larvae expressing mut*TARDBP* resulted in a more vigorous tail thrashing patterns of activity (Fig. 4B,C). To investigate the physiological nature of this altered pattern of activity whole-cell voltage clamp recordings of optogenetically-evoked descending glutamatergic excitatory post-synaptic currents (EPSCs) were recorded in CaP motoneurons. EPSCs recorded in CaP motoneurons expressing mut*TARDBP* revealed that both mean and maximum glutamatergic EPSC were larger in amplitude than synaptic currents recorded in wild type CaP motoneurons (p < 0.05, Fig. 4D–F). Furthermore, optogenetically-evoked glutamatergic EPSCs were also found to occur at a higher frequency in larvae expressing mut*TARDBP* when compared to wild type CaP recordings of EPSCs (p < 0.05, Fig. 4G). The total number of EPSCs recorded over a 5 second exposure to a blue light stimulus in larvae expressing mut*TARDBP* was higher than the total number of EPSCs recorded in CaP motoneurons in either wild type or larvae expressing wt*TARDBP* (p < 0.01, Fig. 4H). This indicates that when driven optogenetically, descending glutamatergic/motoneuron coupling is stronger in the spinal cords of larval zebrafish expressing mut*TARDBP*, suggesting that scaled glutamatergic synapses are functionally stronger following the expression of mut*TARDBP*.

## Discussion

Though considerable advances in our understanding of the genetics of ALS have been made in the past decade, our understanding of spinal cord synaptic defects arising in the disease remains largely unexplored. In this study, we took advantage of the larval zebrafish, a model system that lends itself easily to physiological investigation of spinal cord synaptic function. Building upon previous studies that reported motor impairment^36^ and NMJ synaptic impairment^33,34^, we turned our attention to defects in the descending excitatory synaptic components of the spinal cord motor system of larvae expressing the ALS-associated *TARDBP* missense mutation 1176G > T (encoding the G348C variant). This model has been previously shown to display motor impairment characterized by reduced burst swim durations, distances and swim velocities at 54 hpf^33^. To gain a better understanding of the underlying cause of motor impairment we recorded high-speed videos of touch-evoked tail beat motor patterns in larvae partially embedded in agarose. These experiments revealed that both the total duration of the tail beat and the instantaneous frequency of the tail beat pattern were reduced in larvae expressing mutant *TARDBP* (Fig. 1B–D). Similarly, whole cell patch-clamp recordings of touch-evoked rhythmic muscle EPCs were found to be reduced in amplitude and frequency as well as shorter in burst duration (Fig. 1E,G–I). We have previously argued that much of the impairment in motor function in larvae expressing mut*TARDBP* arises as a result of impaired NMJ synaptic function^33^, and our observation of reduced rhythmic EPC amplitude is in alignment with this theory. However, an argument can be made that reduced tail beat and reduced EPC frequencies arise as a result of an upstream impairment in the neuronal circuit of the locomotor pattern generator in the spinal cord. Studies examining gait rhythm of patients with ALS have revealed that motor function is altered in several ways: 1) the variability in the magnitude of stride-to-stride footsteps is increased and 2) the average stride time is longer and walking speeds are slower^56,57^. The clinical manifestation of ALS is marked by both muscle fatigability and weakness and may account for an increased stride time/reduced walking speed. However, variability in gait rhythm likely has its origins in the locomotor central pattern generating neuronal circuit of ALS patients. We believe that our observations of perturbed locomotor pattern generation in zebrafish expressing mut*TARDBP* could serve as a good model to investigate this poorly understood pathophysiological characteristic of the disease.

Much of the general organization of the vertebrate spinal cord is conserved across species^58,59^ and the development of the motor circuit in zebrafish larvae has been extensively studied (for review see^60–62^). At 2 dpf, larvae display a robust touch response, characterized by vigorous anguilliform burst swimming that, in freely swimming larvae, can last for several seconds^63,64^. Trunk muscles coordinating locomotor behaviour at this stage in development are innervated by three primary, and around twenty secondary motoneurons in each spinal cord hemisegment^65^. Among the primary motoneurons, the CaP motoneuron, which extends its axon to the ventral region of the trunk musculature (Fig. 2A), has been studied previously within the context of this model. Larvae expressing mut*TARDBP* were shown to display impaired NMJ synaptic connectivity with fast-twitch muscle cells characterized by reduced fidelity of NMJ synaptic transmission and reduced EPC amplitudes that were more variable in amplitude when compared to NMJs in larvae expressing wt*TARDBP*^33,34^. In the spinal cord, whole-cell patch-clamp recordings of CaP motoneurons (Fig. 2A,B) expressing mut*TARDBP* were also found to be more excitable in that they displayed a reduced rheobase current and more action potentials were generated upon current injection^33^. In this study, whole-cell patch clamp recordings of touch-evoked CaP motoneuron activity in larvae expressing mut*TARDBP* showed reduced durations of motor activity-related membrane depolarizations and fewer action potentials when compared to our control experimental groups (Fig. 2D,E). Though our subsequent experiments did not focus on the nature of the reduced duration of the tonic membrane depolarizations, it is conceivable that these motoneurons have altered glycinergic synaptic currents. Evidence that reduced glycinergic synaptic transmission could arise in ALS spinal cords has come from a study using a zebrafish *Sod1*^G93R^ model that reported a decrease in quantal release frequency of glycinergic mEPSC in CaP motoneurons at 4 dpf, at a time when these neurons were shown to mount a stress response^66^. Moreover, *in vitro* motoneuron patch-clamp recordings from dissociated spinal cord cultures of *SOD1*^G93A^ transgenic mice have revealed reduced amplitudes of glycinergic miniature inhibitory post-synaptic currents^67^. Taken together, dysregulation of inhibitory components of the motor system that tip the excitatory/inhibitory balance of synaptic inputs in favour of excitation could play a role in excitotoxicity (for review see^68^).

A general observation made in our recordings of CaP motoneurons following touch-evoked fictive motor patterns was the presence of increased spontaneous excitatory post-synaptic potentials (Fig. 2B insets). To investigate the nature of these events more accurately, we isolated and recorded AMPA/kainate-mediated glutamatergic mEPSCs in CaP motoneurons (Fig. 3A) and found that in addition to occurring at a higher frequency, the quantal size of these events was also significantly larger in larvae expressing mut*TARDBP* (Fig. 3B,C). The increase in mEPSC frequency is similar to what has been observed in *in vitro* slice recordings of layer V pyramidal neurons from the motor cortex of pre-symptomatic mice expressing the ALS-associated *TARDBP*^Q331K^ variant, which show an increase in spontaneous EPSC frequency when compared to recordings derived from wild type animals^31^ but differ from *in vitro* slice recordings derived from *TARDBP*^A315T^ mice which display reduced EPSC frequencies^69^. When we examined the entire population of mEPSCs, plotted as a cumulative histogram, a right-shifted skewing of the curve from mut*TARDBP*-expressing larvae was observed indicating synaptic scaling (Fig. 3D). Synaptic scaling likely results from the insertion of additional AMPA receptors at excitatory synapses in CaP motoneurons. Changes in glutamate receptor abundance are intimately linked with homeostatic synaptic plasticity^70^ and we believe that this is relevant for ALS pathophysiology. Homeostatic synaptic plasticity orchestrates the long-term stabilization of neuronal circuit function in spite of destabilizing biotic and abiotic influences. Dysfunction in mechanisms that regulate homeostatic synaptic plasticity has been implicated in several pathologies, including autism spectrum disorder, mental retardation, psychiatric disorders, epilepsy and Alzheimer’s disease^71–78^. Certain aspects of ALS pathology could manifest as a result of defective or augmented cellular pathways that normally coordinate synaptic plasticity. Generally, a neuron’s response to perturbations can be divided into three areas: the homeostatic control of neurotransmitter release, receptor expression/synaptic scaling, and control over the intrinsic excitability of a cell (for review see^79–81^). Alterations in any or all of these may arise in the ALS spinal cord but have not been explored in animal models of TDP-43.

One possibility is that before neurodegeneration occurs in the ALS spinal cord, increased and deleterious descending glutamatergic synaptic activity drives a maladaptive homeostatic compensatory change in lower motoneurons. Alternatively, or perhaps occurring in concert, impaired functioning of TDP-43 ALS-causing variants and/or loss of nuclear localization of TDP-43 could perturb the processing of transcripts that are involved in coordinating cellular processes that stabilize neuronal function following perturbations. Though we were unable to address these questions in the present study, we were able to take advantage of the unsurpassed optical transparency of zebrafish larvae and optogenetically drove locomotor behaviour by expressing channelrhodopsin in the hindbrain and spinal cord population of *Chx10* expressing (Fig. 4A) glutamatergic V2a class of interneurons^53^. These neurons have ipsilateral descending axon projections that form *en passant* excitatory synapses with spinal cord motoneurons. As with our touch-evoked motor behaviour, optogenetically driving activity in this population of glutamatergic interneurons could reliably evoke swimming behaviour in both wild type and larvae expressing wt*TARDBP* (Fig. 4B,C). However, unlike the reduced touch-evoked motor behaviour observed in mut*TARDBP-*expressing larvae, optogenetically driving motor activity in these animals resulted in uncoordinated thrashing of the tail indicating that descending excitatory inputs to spinal cord motoneurons are functionally altered (Fig. 4B,C). Whole-cell patch clamp recordings of EPSCs in CaP motoneurons during optogenetic stimulation of *Chx10* interneurons revealed that EPSCs were larger in both mean and maximal amplitudes in larvae expressing mut*TARDBP* (Fig. 4E,F) and more EPSCs were evoked at a higher frequency (Fig. 4G,H) when compared to wild type larvae. Part of the increase in amplitude of EPSCs likely arises as a result of the increase in quantal size observed in larvae expressing mut*TARDBP*. We also believe that the increase in frequency of mEPSCs observed in larvae expressing mut*TARDBP* may indicate that the readily releasable pool of synaptic vesicles at these excitatory synapses is larger and may also partially account for the increase in EPSC amplitude and frequency when these synaptic connections are optogenetically forced to fire. Though the exact molecular mechanisms underlying the change in connectivity between descending excitatory glutamatergic interneurons and spinal motoneurons in our model remains to be uncovered, it is clear that this is an understudied area of ALS research and worthy of further investigations.

## Supplementary information

Supplementary Material, Video S1

Supplementary Material, Video S2

Supplementary figure

## Data Availability

The datasets generated during and/or analysed during the current study are available from the corresponding author on reasonable request.
