# Supplementary material for: Augmentation of spinal cord glutamatergic synaptic currents in zebrafish primary motoneurons expressing mutant human TARDBP (TDP-43)
Source: Sci Rep. 2019 Jun 24;9:9122. doi: 10.1038/s41598-019-45530-3 (PMC6591224; doi:10.1038/s41598-019-45530-3)
Supplement: Supplementary file 3 — Supplementary figure [file 41598_2019_45530_MOESM3_ESM.docx]

Augmentation of spinal cord glutamatergic synaptic currents in zebrafish primary motoneurons expressing mutant human *TARDBP* (TDP-43).

Virginie Petel Légaré*, Ziyaan A. Harji*, Christian J. Rampal, Xavier Allard-Chamard, Esteban C. Rodríguez and Gary A.B. Armstrong

**Supplementary files**

**Supplementary Material, Figure S1**


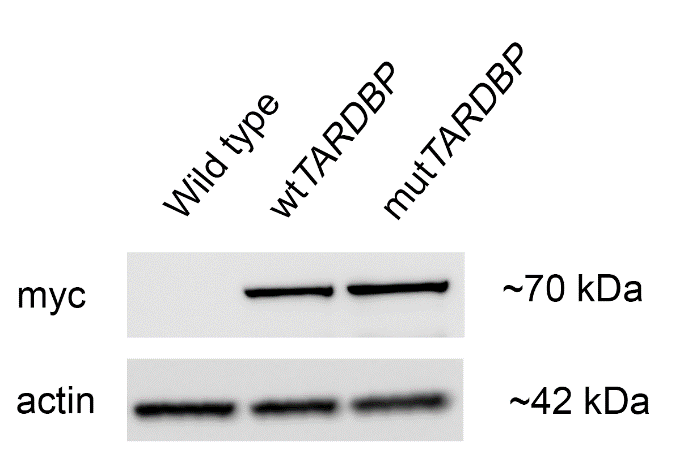


Western blot displaying wild type and mutant TDP-43 expression levels in 40 pooled larvae aged 54 hpf from each treatment group. The high molecular weight results from the addition of a N-terminal FLAG-tag and 6 C-terminal myc-tags to TDP-43. Actin was used as a loading control.

**Supplementary Material, Video S1**

Touch-evoked motor behaviour in wild type 2 day old zebrafish larva, larva expressing wt*TARDBP*, and larva expressing mut*TARDBP* mRNA. Video sequences are representative touch-evoked motor behaviour recorded in black and white and captured at 200 frames per second (fps).

**Supplementary Material, Video S2**

Optogenetically-evoked motor behaviour in transgenic, Tg(*Chx10:ChRWR-EGFP*), 2-day old zebrafish larvae expressing wt*TARDBP* and mut*TARDBP* mRNA. Video sequences are representative of optogenetically-evoked motor behaviour recorded in black and white and captured at 120 fps. Videos contain a 5 second exposure to blue light stimuli (dimming of video).
